# MUC5B Polymorphism and Susceptibility to Idiopathic Pulmonary Fibrosis in Morocco

**DOI:** 10.7759/cureus.86806

**Published:** 2025-06-26

**Authors:** Lamiyae Senhaji, Meriame Abbassi, Nadia Senhaji, Soumaya Benmaamar, lamyae Dani, Karima El Rhazi, Meryem Karhate, Mounia Serraj, Mohamed El Biaze, Mohammed Chakib Benjelloun, Karim Ouldim, Laila Bouguenouch, Bouchra Amara

**Affiliations:** 1 Department of Pulmonology, Hassan II University Hospital Center, Fez, MAR; 2 Epidemiology and Research in Health Science Laboratory, Faculty of Medicine, Pharmacy, and Dental Medicine, Sidi Mohamed Ben Abdellah University, Fez, MAR; 3 Biomedical and Translational Research Laboratory, Faculty of Medicine, Pharmacy, and Dental Medicine, Sidi Mohamed Ben Abdellah University, Fez, MAR; 4 Department of Genetics, The Higher Institute of Nursing Professions and Health Techniques, Fez, MAR; 5 Department of Biology, Faculty of Sciences, Moulay Ismail University, Meknes, MAR; 6 Department of Epidemiology and Public Health, Sidi Mohammed Ben Abdellah University, Fez, MAR; 7 Department of Medical Genetics and Oncogenetics, Hassan II University Hospital Center, Fez, MAR

**Keywords:** africa, genetics, idiopathic pulmonary fibrosis, morocco, muc5b

## Abstract

Background: Idiopathic pulmonary fibrosis (IPF) is a chronic, progressive interstitial lung disease of unknown etiology. It has been demonstrated in European, American, and Asian populations that genetic factors, particularly the MUC5B rs35705950 SNP, represent a significant risk factor for the development of this disease. To date, no studies have been performed within African populations, thereby necessitating the execution of the present investigation.

Methods*:* A case-control study was conducted from September 2021 to May 2024. It included 55 patients diagnosed with IPF and 61 control subjects. Comprehensive demographic, clinical, radiological, functional, and therapeutic data were collected. Additionally, genetic analysis for the MUC5B rs35705950 SNP was carried out in the genetic department of the University Hospital Hassan II of Fez (Morocco).

Results*:* The patient cohort predominantly consisted of men, with a sex ratio of 49 men to six women. The mean age was 67.72 ± 6.406 years. Of the subjects, 58.2% were former smokers. Dyspnea was the most prevalent symptom (89.1%). Radiological assessments revealed that 70.9% of patients exhibited definite usual interstitial pneumonia. The median survival time was recorded at 4.75 years. Statistical analysis indicated a significant association between the MUC5B rs35705950 SNP and IPF (p = 0.0001).

Conclusions*:* This study confirms that the MUC5B rs35705950 SNP is a major risk factor for IPF within the African population. It represents the first investigation of its kind on the African continent, with the hope that it will motivate further research efforts in Africa to yield more representative data.

## Introduction

Idiopathic pulmonary fibrosis (IPF) is classified as a chronic interstitial lung disease (ILD) predominantly affecting adults aged 50 to 70 years, with a notable male predominance, comprising approximately 70% of cases [1]. The incidence of IPF is estimated to range from 3 to 10 cases per 100,000 individuals per year within American and European populations [2]. However, specific epidemiological data for African populations remain scarce. A multiethnic study conducted in Paris indicated a higher prevalence of IPF among North African individuals compared to their European counterparts, with rates of 26.9 versus 5.8 cases per 100,000, respectively [3]. Genetic predisposition is suggested by findings that indicate 2% to 20% of patients with IPF have at least one first-degree relative diagnosed with diffuse infiltrative lung disease [4]. Numerous studies conducted on Caucasian and Asian populations have investigated the association between genetic variations and IPF risk factors [5-7]. These studies have identified various polymorphisms and genetic mutations correlated with IPF; however, the MUC5B promoter variant rs35705950 is the most strongly associated risk factor [8-13]. In light of the limited research concerning the African population, the present study aims to elucidate the characteristics of IPF within this demographic and to assess the prevalence of the MUC5B polymorphism in the African context. No similar studies were conducted in other African populations. This study represents the first of its kind on the continent, with the objective of contributing to a better understanding of IPF in African populations.

## Materials and methods

Study design

The present investigation is a case-control study conducted at the Hassan II University Hospital in Fez, Morocco. It spanned nearly three years, from September 2021 to May 2024.

All patients diagnosed with IPF at the Hassan II University Hospital were included in the study, regardless of age. The diagnosis was established following a multidisciplinary discussion, ensuring that each patient underwent a comprehensive assessment. Patients who declined to participate in the study were excluded from the analysis. In total, 55 patients with IPF were enrolled, alongside a control group comprising 61 individuals, who were matched to cases by age and sex, and included hospitalized patients from other departments and hospital staff, all of whom exhibited no chronic pulmonary diseases or familial history of ILD to minimize bias.

Demographic, clinical, radiological, functional, therapeutic, and genetic data were meticulously collected and recorded in an Excel spreadsheet. These data were subsequently coded and analyzed using IBM SPSS Statistics for Windows, Version 26 (Released 2019; IBM Corp., Armonk, New York, United States) within the Epidemiology and Public Health Laboratory of the Faculty of Medicine, Pharmacy, and Dental Medicine of Fez.

Informed written consent was obtained from all participants after they were thoroughly briefed on the study's objectives. Participation was entirely voluntary. Additionally, ethical approval for the study was granted by the University Hospital Ethics Committee of Fez, under reference number 03/22.

Genetic screening

Peripheral blood samples (10 mL) were obtained from all recruited patients using EDTA tubes. Genomic DNA was subsequently extracted employing the Invitrogen™ PureLink™ Genomic DNA Mini Kit. The quantification of the extracted DNA was performed using a Nanodrop spectrophotometer (Nanodrop; Fisher Scientific, Wilmington, DE, USA).

To screen for MUC5B rs35705950 SNP, polymerase chain reaction (PCR) was conducted utilizing the following primers: Muc5B-F: 5′‐TGATCACTGGTGCCTGGACG‐3′ and Muc5B-R: 5′‐ACACAGTGACACCAAACAAGTG‐3′. The PCR reactions were prepared in a final volume of 25 µL, consisting of 1 µL of genomic DNA (100 ng), 10 µL of DreamTaq Green PCR Master Mix (2x) (Invitrogen), 1 µL of each primer (10 µmol/L), and 12 µL of nuclease-free water.

The PCR amplification protocol included an initial denaturation step at 95 °C for three minutes, followed by 35 cycles of denaturation at 95 °C for 30 seconds, annealing at 58 °C for three minutes, and extension at 72 °C for one minute. A final extension step was performed at 72 °C for nine minutes.

Post-amplification, the PCR products were purified using ExoSAPIT™ (USB Corporation, OH, USA) and subsequently sequenced with BigDye Terminator v3.1. Capillary electrophoresis was executed on the Applied Biosystems 3500Dx Genetic Analyzer (Applied Biosystems, CA, USA). The resulting chromatograms were analyzed using the Sequencing Analysis Software (SeqA v.5.4; Applied Biosystems). The sequences obtained underwent bioinformatics analysis via the “Nucleotide Blast” alignment program [14].

Statistical analysis

Quantitative variables are presented as means, while standard deviations and qualitative variables are expressed as percentages. For the multivariate analysis, the Chi-squared (χ²) test and Fisher test were employed to compare percentages, and Student's t-test was utilized for the comparison of means.

Survival analysis was conducted using the Kaplan-Meier method to estimate survival rates. A significance level of 0.05 was established for all statistical tests.

## Results

Demographic, clinical, radiological, and functional characteristics of the patients diagnosed with IPF are summarized in Tables 1, 2.

**Table 1:** Demographics and smoking habits of IPF patients and controls IPF: Idiopathic pulmonary fibrosis

Variable	IPF (n=55)	Controls (n=61)	p	Chi-square value
Age	67.72+/-6.406	66.92+/-9.459	0.599	0.277
Gender (M/F) n	49/6	53/8	0.716	0.714
Smoking habits (n)				
Non-smoker	19	19	0.092	0.136
Former smoker	32	44		
Current smoker	4	8		

**Table 2:** Clinical, radiological, and functional characteristics of IPF patients IPF: Idiopathic pulmonary fibrosis; UIP: usual interstitial pneumonia; FVC: forced vital capacity

Variable	IPF (n=55)
Professional exposure n (%)	14(25.5%)
Comorbidities n (%)	19 (34.5%)
Onset of symptoms (months)	29.11
Symptoms n (%)	
Cough	46 (83.6%)
Dyspnea	49 (89.1%)
Chest pain	7 (12.7%)
Hemoptysis	5 (9.1%)
Physical examination	
SaO2 (mean)	94.38% (+/-4.03%)
Crackles	48 (87.3%)
Cubbing sign	19 (34.5%)
Radiological features n (%)	
Definite UIP	39 (70.9%)
Probable UIP	3 (5.5%)
Indeterminate to UIP	13 (23.6%)
FVC%	64 (+/-18.17)
Severe restriction n (%)	13 (23%)
Treatment (Y/N)	22/33
Evolution (respiratory function) n (%)	
Worseness	15 (27.3%)
Stability	18 (32.7%)
Death n (%)	4 (7.27%)

Demographic and clinical features

The patient cohort predominantly consisted of male patients, with a sex ratio of 49/6. The mean age of the participants was 67.72 ± 6.406 years (from 49 to 85). Among the subjects, 58.2% were former smokers and 7.3% were current smokers. Additionally, 25.5% reported professional exposure to potential lung irritants. In three cases, a tumor was identified in association with IPF, specifically two cases of pulmonary adenocarcinoma and one case of epidermoid carcinoma of the cavum. Comorbidities were present in 34.5% of the patients, with the most common conditions being diabetes, hypertension, and cardiopathy.

The mean duration from the onset of symptoms to consultation was 29.11 months, a delay that may be attributed to the advanced age of the patients, who often ascribed their dyspnea to the aging process. Dyspnea was the most prevalent symptom, reported by 89.1% of the cohort. During physical examination, 28.3% of patients exhibited oxygen saturation levels below 90%, indicative of chronic respiratory failure.

Radiological evaluations revealed that 70.9% of the patients exhibited definite usual interstitial pneumonia (UIP), while 23.6% presented with an indeterminate pattern for UIP.

Only 22 patients (40%) were receiving treatment with nintedanib, primarily due to the unavailability of pirfenidone in the country and the prohibitively high cost of nintedanib, which is not fully covered by all health insurance companies.

The subjects were monitored for a mean duration of 16 months, with a range of 1 to 59 months. Notably, 16.36% of participants were lost to follow-up, and four patients (7.27%) succumbed during the study period. The median survival time was calculated at 4.75 years; at the three-year mark, 80% of patients remained alive, whereas at five years, only 40% were still living.

Genotype distribution

The distribution of genotypes for each SNP in patients with IPF and the control group is presented in Table 3.

**Table 3:** Genotypes distribution of MUC5B in IPF patients and controls IPF: Idiopathic pulmonary fibrosis

	Genotypes		
MUC5B			
rs35705950	GG n (%)	GT n (%)	TT n (%)
IPF	17 (30.9)	33(60)	5(9.1)
Controls	47(77)	14(23)	0
P/chi-square value	0.0001/ <0.00001		
rs2672794	CT %	CC %	TT %
IPF	19 (34.5)	36 (65.5)	0
Controls	24 (39.3)	33 (54.1)	4 (6.6)
P/chi-square value	0.140/ 0.125		

The MUC5B rs35705950 SNP demonstrated a statistically significant association with IPF, with a higher frequency observed among IPF patients compared to controls (Pearson's Chi-squared test: p = 0.0001).

Conversely, no significant difference was identified between IPF patients and controls for the MUC5B rs2672794 SNP.

Associations

No SNP genotype was found to be significantly associated with gender, body mass index (BMI), or comorbidities. However, the CC genotype of the MUC5B rs2672794 SNP was significantly more prevalent among smokers (chi-squared test: p = 0.01). In contrast, no associations were observed between any genotypes of MUC5B rs35705950 and smoking habits.

In terms of radiological findings, a significant relationship was identified between the presence of the MUC5B rs35705950 SNP and the radiological diagnosis of IPF, which did not necessitate histological confirmation or additional investigations (chi-squared test: p= 0.043). Specifically, among the 36 patients exhibiting a definite or probable UIP pattern on computed tomography (CT), the MUC5B rs35705950 SNP was present.

No significant relationship was identified between the MUC5B SNPs and forced vital capacity (FVC), nor with the distance covered in the six-minute walk test.

Disease progression was evaluated based on clinical data as well as lung function metrics. No decline over time in disease severity was found to correlate with the genotypes of either MUC5B rs35705950 or MUC5B rs2672794.

No significant associations were observed between the presence of the MUC5B rs35705950 or MUC5B rs2672794 genotypes and patient survival.

Acute exacerbations occurred in 11 out of 55 patients (20%). This exacerbation revealed the disease among a subset of patients. Fisher's exact test indicated no significant association between the MUC5B rs35705950 or MUC5B rs2672794 SNPs and the occurrence of acute exacerbations.

## Discussion

IPF has multiple risk factors, among which genetic determinants are recognized as significant contributors to disease susceptibility. The MUC5B polymorphism is the most extensively studied genetic variant associated with an increased risk of developing IPF [8-13].

Mucin 5B, a mucin secreted by the bronchial glands, constitutes a major component of mucus in the respiratory tract. It plays a critical role in mucociliary clearance, infection control, and immune regulation [15,16]. The MUC5B polymorphism results in the overexpression of mucin 5B in the bronchioles and alveoli. This chronic hypersecretion and accumulation of mucus within the airspaces impair mucociliary transport, leading to mucus adhesion, chronic inflammation, and subsequent tissue injury [17]. Furthermore, the presence of honeycomb cysts in IPF exhibits intermediate characteristics of ciliated and distal epithelial phenotypes, suggesting aberrant differentiation and a failure of regenerative processes following injury [18-20].

Overall, the MUC5B polymorphism is a crucial factor in the pathogenesis of IPF, accounting for approximately 30-35% of individuals who develop the disease [10]. Additionally, this genetic variant may serve as a predictive marker for IPF, facilitating the early detection of asymptomatic cases of preclinical pulmonary fibrosis in at-risk populations, such as smokers and families with multiple cases of pulmonary fibrosis [15,21].

Given the significant implications of the MUC5B polymorphism, it is essential to investigate its prevalence and impact within our population. As this study represents the first of its kind within the African context, it is crucial to determine whether our demographic, clinical, radiological, and functional data are comparable to findings from European, American, and Asian studies before delving deeper into the implications of the MUC5B mutation.

Initial analyses indicate that the demographic characteristics of our study align with those of other research studies. Notably, the mean age of IPF patients in our study was 67 years, compared to 63 years in the study by Bonella et al. (Germany) [22], 70 years in the study by Biondini et al. (Italy) [23], and 66.7 years in the Indian study conducted by Dhooria et al. [24]. Furthermore, a consistent pattern of male predominance was observed, with respective proportions of 89%, 77.4%, and 81% in our study, the Indian study, and the Italian study. A recent meta-analysis conducted by Singh et al. in 2024 [25], which encompassed 52 studies from European, American, Mexican, and Asian populations totaling 13,652 patients with IPF, corroborated these findings, reporting a mean age of 64.72 years and a male-to-female ratio of 2.27. Additionally, smoking was identified as the primary risk factor across our study and others [22-24], while comorbidities, particularly cardiovascular conditions, were noted in all studies [23,24].

In our study, CT scans were sufficient to establish a diagnosis of IPF without the need for histological confirmation in 76.4% of patients. This is notably lower than the findings from an Indian study, which reported a diagnostic rate of 93% based solely on CT imaging, and an Italian study that found a rate of 90.9% [23,24].

All studies, including ours, demonstrated that patients exhibited moderate restrictive patterns on spirometry, with median FVC percentages reported as 64% in our cohort, 69.2% in the Indian study [24], and 69% in the German study [22]. In contrast, the Italian study reported a higher baseline FVC of 77% [23].

The median survival of our patient population was 4.75 years, which is at the upper limit of what is documented in the literature but still below the 5.9 ± 3.2 years reported in the German study [22] and the Italian study, where the five-year survival rate exceeded 70% [23]. This discrepancy may be attributed to the limited number of patients in our cohort receiving antifibrotic therapy.

Regarding the MUC5B polymorphism, we confirmed that the MUC5B rs35705950 SNP is associated with IPF, a finding consistent with several previous studies [22,23,24,26,27]. A meta-analysis conducted by Singh et al. [25] examined the relationship between MUC5B rs35705950 and susceptibility to IPF across 18 studies from diverse continents, including Europe, America, and Asia, encompassing 6,161 patients and 13,556 controls. This analysis revealed a robust association between the rs35705950 variant and an increased risk of IPF. Additionally, subgroup analyses by ethnicity indicated a stronger association for the risk allele among Europeans (T vs. G, odds ratio [OR]: 4.05) compared to Asians (T vs. G, OR: 2.54). Other meta-analyses have similarly emphasized the significant relationship between the T allele and IPF susceptibility [27-29]. However, our study did not observe this association, likely due to the small sample size.

Furthermore, we found no association between the MUC5B rs2672794 SNP and susceptibility to IPF. This particular SNP has been infrequently associated with IPF and is primarily linked to the risk of pneumoconiosis among coal workers [30].

In our study, no significant differences were observed regarding gender, smoking status, or functional characteristics between carriers and non-carriers of the minor allele T (MUC5B rs35705950). Similar findings have been reported by Borie et al. and Bonella et al. [8,22].

Notably, our analysis indicated that the MUC5B rs35705950 polymorphism was associated with a higher proportion of definitive radiological diagnoses, specifically UIP and probable UIP (p = 0.043). This finding aligns with the results of Chung et al. [31] but contrasts with the cohort studied by Biondini et al., where the T allele was associated with a lower percentage of radiological diagnoses [23].

Clinically, the MUC5B polymorphism has been linked to cough severity, likely due to its role in the overproduction and accumulation of mucin in the distal airways, which can impair mucociliary function and trigger cough [32]. However, in our study, we did not find a significant relationship between cough severity and the presence of this polymorphism.

Functionally, no notable differences were detected among the various genotypes (TT, GT, or GG). This finding is consistent with the results of van Der Vis et al., who reported no association between the MUC5B minor allele and the severity of lung function at diagnosis in both sporadic and familial IPF cases [33]. Conversely, Biondini et al. [23] and Peljto et al. [34] described differences in FVC, although they did not specify whether these differences were statistically significant.

In terms of survival, multiple studies have confirmed an association between the MUC5B rs35705950 SNP and improved survival outcomes [24,34,35]. However, some studies have refuted this, indicating increased mortality among patients carrying this polymorphism [36]. Our findings, similar to those of van der Vis et al. [33], did not establish a relationship between the presence of the minor allele and survival. A recent statistical analysis aimed at reassessing the association of MUC5B with survival in IPF, utilizing contemporary methods and datasets, concluded that there is no robust evidence supporting index event bias or a significant association between MUC5B and either increased or decreased survival [37].

While this study provides initial insights into the relationship between MUC5B and IPF, several limitations warrant cautious interpretation of the findings. The relatively small sample size may limit the statistical power to detect more subtle associations or to generalize the results to a broader population with IPF. Furthermore, the monocentric design means that the findings might be specific to the patient cohort and clinical practices of the single institution involved, potentially overlooking variations present in other centers or demographic groups. Lastly, the short duration of follow-up restricts our understanding of the long-term implications of MUC5B in the progression of IPF and its impact on ultimate patient outcomes.

## Conclusions

This research represents a pioneering investigation on the African continent and offers valuable insights into genetic studies across diverse ethnicities, thereby mitigating the effects of population stratification and enhancing the robustness of our findings. Therefore, we regard our study as an initial step that lays the groundwork for future research aimed at validating our findings in larger, more diverse populations.
